# Advances in the application of patient-derived xenograft models in acute leukemia resistance

**DOI:** 10.20517/cdr.2025.18

**Published:** 2025-05-28

**Authors:** Ronghao Qin, Yuxing Liang, Fuling Zhou

**Affiliations:** Department of Hematology, Zhongnan Hospital of Wuhan University, Wuhan 430072, Hubei, China.

**Keywords:** Patient-derived xenografts, acute leukemia, leukemia stem cells

## Abstract

Acute myeloid leukemia (AML) and acute lymphoblastic leukemia (ALL) are genetically heterogeneous malignancies of hematopoietic stem cells, characterized by complex mutations and a high risk of drug resistance and relapse. Patient-derived xenograft (PDX) models are dynamic entities transplanted with leukemia stem cells (LSCs), retaining patients’ biological and genetic characteristics. By elucidating LSCs, clonal dynamics, and microenvironment interaction, PDXs facilitate the preclinical evaluation of therapy sensitivity, including immunotherapies, epigenetic therapies, and other agents targeting mutated proteins or apoptosis. The application of PDXs has provided translational evidence for various studies with reliable clinical relevance. Additionally, conventional PDXs remain a robust tool in identifying drug resistance compared with other models, and their potential is further unleashed when examined in large cohorts or combined with novel technologies, which not only enhances our understanding of acute leukemia biology but also enables the discovery and identification of novel biomarkers. In this review, we present the application of PDX models for acute leukemia resistance, including mechanism investigation, therapy evaluation, and associated challenges.

## INTRODUCTION

Acute myeloid leukemia (AML) and acute lymphoblastic leukemia (ALL) are highly heterogeneous hematological malignancies distinguished by the clonal disorder of myeloid or lymphoid blasts in the bone marrow^[1]^. Over the past decades, our understanding of acute leukemia’s complex genomic profile and evolving clonal nature has expanded extensively^[2]^. However, current knowledge is still inadequate for fully handling leukemia resistance, and countless patients could benefit from novel therapy and tailored drug combinations. Therefore, a preclinical model that accurately recapitulates the original characteristics of acute leukemia for therapy evaluation is necessary.

Previous studies have delved into a multitude of preclinical models, encompassing *in vitro* models, genetically engineered models (GEMs), cell-line-derived xenograft models (CDXs), and patient-derived xenograft models (PDXs). Among the preclinical models, PDX models are widely recognized as the most clinically relevant^[3]^. They involve implanting leukemia stem cells (LSCs) into immunocompromised mice. PDXs closely resemble the inter- and intra-tumor heterogeneity observed in patients and provide valuable insights into the pathobiology of acute leukemia, including the role of the bone marrow microenvironment in disease progression and therapy resistance^[4]^. Furthermore, PDXs have been the practical and reliable choice for screening and discovering novel therapeutic targets, propelling precision medicine in acute leukemia treatment^[5,6]^.

Recent advancements in immunodeficient modifications and humanized techniques have further enhanced the utility of PDX models, enabling better recapitulation of the molecular and biological features of primary tumor samples. When combined with the lentiviral vector, RNA interference, and CRISPR/Cas9 genome editing technology, PDXs are crucial in investigating how specific mutations affect leukemia behavior and response to therapies, providing unprecedented precision and control in cancer research^[7,8]^. Moreover, injecting hematopoietic or immune cells alongside leukemia cells has demonstrated remarkable potential in predicting immunotherapy outcomes and uncovering the interaction between leukemia and the hematopoietic system^[9]^. Despite these advances, there remain challenges in fully harnessing the potential of PDX models. Factors such as variations in engraftment rates, degrees of clinical consistency, and the limited time window for evaluating aggressive AML patients hinder their widespread clinical application.

In this review, we delineated how PDXs mirror aspects of acute leukemia, including initiation, clonal evolution, and microenvironment interaction. Additionally, we present the resistance mechanism indicated by the PDX experiment. Finally, we highlighted the preclinical evaluation of targeted agents, immunotherapy, and other novel therapies against acute leukemia. By bridging the gap between preclinical research and clinical application, PDX models play a crucial role in investigating drug resistance, validating resistance targets, conducting drug screening, and providing a unique opportunity to tailor acute leukemia treatment. This work explores the pivotal role of the PDX model in unraveling the mechanisms of acute leukemia resistance and illuminates the future potential of humanized models in developing therapeutics against acute leukemia.

## FACTORS RELATED TO THE ESTABLISHMENT OF PDXS

**Figure 1:**
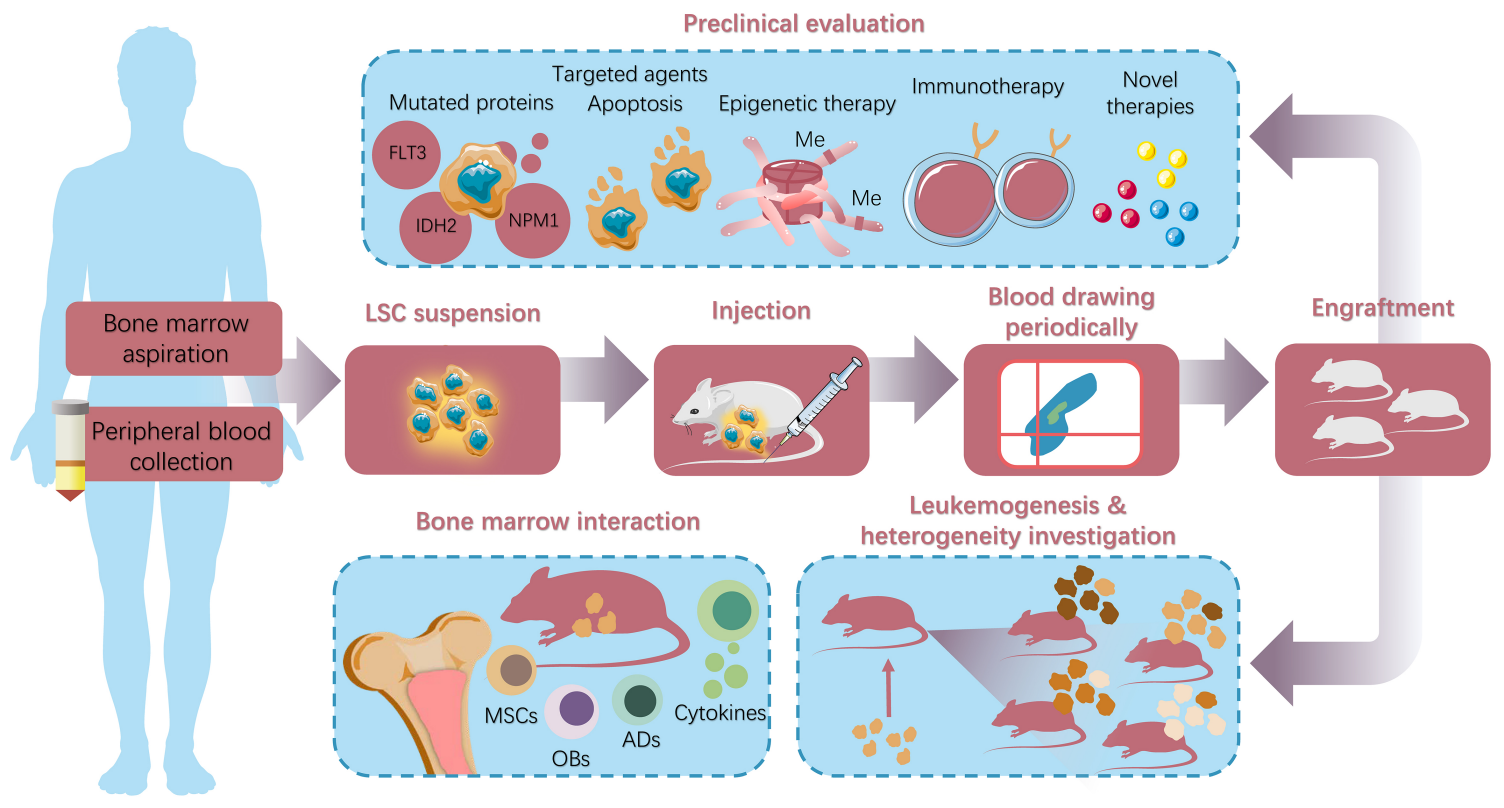
The establishment and application of acute leukemia PDXs. The general process of constructing the acute leukemia PDX model involves sample collection (according to immunophenotypes or mononuclear cell population), pretreatment (if necessary), injection into immunodeficient mice, and subsequent engraftment monitoring. The well-accomplished engraftment enables further studies on acute leukemia, including LSCs, clonal dynamics, heterogeneity, and microenvironment interaction involving MSCs, OBs, ADs, and relative cytokines. In preclinical experiments, PDXs are invaluable tools for identifying therapy sensitivity in translational research. The PDX model has been proven to be a significant platform for evaluating various acute leukemia therapies, including targeted agents, immunotherapies, and other novel therapies. Adapted from Smart Servier Medical Art (https://smart.servier.com), licensed under CC BY 4.0; annotations and color modifications were added by the authors using Adobe Illustrator 2024. PDXs: Patient-derived xenografts; LSCs: leukemia stem cells; MSCs: mesenchymal stem cells; OBs: osteoblasts; ADs: adipocytes.

The conventional construction procedure isolates leukemia cells from bone marrow or peripheral blood samples and then transplants them into preconditioned immunodeficient mice. To identify the engraftment, flow cytometry, bone marrow smears, and peripheral blood smears ought to be applied [Figure 1]. To establish a reliable PDX model, we should not only carefully study the relevant protocols but also make adjustments according to our investigation. The main elements that may change the comfort zone of PDXs, such as pretreatment, injection site, and mouse strain, are listed in the Supplementary Materials.

## RESISTANCE MECHANISM: MODELING LEUKEMOGENESIS

### LSCs in the PDX model

LSCs have been recognized as both a potential cause of resistance and a therapeutic target. By simulating leukemia development and supporting leukemia cell proliferation across diverse risk strata, the PDX platform offers unique insights into the nature of these cell populations. Researchers managed to identify the initiation subgroups of AML cells in AML PDXs^[10]^ and found that leukemogenesis can be promoted by interleukin-3 (IL-3), granulocyte-macrophage colony-stimulating factor (GM-CSF), and stem cell factor (SCF)^[11]^. Interestingly, the LSC abundance in PDXs was found to be correlated with the clinical severity and prognosis of patients^[12,13]^.

The precise recreation of LSCs in PDX models presents a pivotal opportunity to delve deeply into their distinctive characteristics and potential therapeutic vulnerabilities. Within AML specimens, LSCs merely constitute a minority^[10,14]^, significantly fewer than pre-leukemic hematopoietic stem cells (HSCs)^[15]^, but their self-renewal capability contributes greatly to the progression and relapse of leukemia^[13,16]^. Since LSCs behave distinctly differently from non-LSCs^[16]^, one critical question arises: How do we effectively discern LSCs from the multitude of distinct subpopulations in acute leukemia? Researchers isolated cell subsets based on surface markers or performed genomic editing and monitored their *in vivo* behavior^[17]^. The uncontrolled myeloid or lymphoid blast proliferation indicates the presence of LSCs in the transplanted subset. Conversely, multilineage human hematopoiesis reconstitution suggests the subset contains normal HSCs or pre-leukemic stem cells^[18]^. Furthermore, the integration of humanized niches in studying acute promyelocytic leukemia (APL) highlighted that LSCs were not exclusively confined to HSCs or multipotent progenitors but also resided within lineage-committed progenitors^[19]^.

Moreover, PDXs have illuminated the phenotypic diversity of LSCs in AML, with examples including CD34+^[20]^, CD117+^[21]^, CD38+, and lineage markers-positive (Lin+)^[14]^, while LSCs in ALL tend to occupy higher frequencies of leukemia cells and exist across various stages of differentiation^[22,23]^. These findings emphasize that LSCs do not solely occupy a single subgroup, and PDXs are useful for further exploration.

Identifying signaling pathways and biomarkers specific to LSCs using PDXs has deepened our comprehension of acute leukemia pathogenesis and facilitated drug discovery efforts. For instance, researchers performed secondary transplantation and found that targeting CD44 eliminated LSCs^[24]^. González-García *et al.* identified the increasing expression of interleukin-7 receptor (IL-7R) in ALL initiation through serial passages^[17]^. Furthermore, the dynamic evolution of LSCs under treatment pressure underscores the existence of distinct subgroups that may play pivotal roles in drug resistance. In PDXs with suboptimal response to cytarabine (Ara-C), the fraction of calcitonin receptor-like receptor-positive (CALCRL+) AML surged significantly following Ara-C treatment, and suppressing CALCRL expression markedly diminished the prevalence of LSCs, suggesting a link between CALCRL expression and drug resistance^[25]^. Comparatively, in AML PDXs treated with venetoclax-azacitidine, researchers characterized a unique LSC subclass that demonstrated resilience and was responsible for monocytic disease progression in patients with AML treated with venetoclax-based regimens^[26]^.

### Clonal dynamics in the PDX model

The PDX model provides insights into potential resistance targets by maintaining the genetic heterogeneity of multiple clones and modeling the evolving genetic landscape over time. Currently, faithfully copying the continuous clonal evolution of leukemia cells in the clinical situation seems a little far-fetched. However, PDXs remain a robust tool when we strike a balance between our research needs and the number of passages: limited passages reflect the primary traits of acute leukemia more accurately, while serial passaging offers a deeper understanding of the clonal evolution and selection process that occurs during treatment, presenting you distinctive characteristics of various subgroups^[27]^. Recent studies utilizing advanced sequencing and labeling technology have documented the occurrence of clonal competition and illuminated the intricate patterns of clonal evolution in acute leukemia PDXs [Table 1].

**Table 1:** Acute leukemia PDX model in investigating clonal dynamics

**Ref.**	**Mouse strain**	**Model counts**	**Patient counts**	**Subject**
[28]	NOG	105	160	Recurrent or refractory AML clones exhibit biased engraftment in 57% of the models, despite having a VAF below 5% in the patients Patients whose cells successfully engrafted in PDX models exhibit significantly lower event-free survival rates than those whose cells failed to engraft
[29]	NSG-SM3	7	3	Verify the development and evolutionary potential of AML clones *in vivo*, including cases of convergent evolution and both branching and linear evolutionary processes
[30]	NSG	119	160	Genetic variation frequencies in PT and PDX samples are largely correlated, while there are some noticeable differences as well, especially when the VAF is low
[18]	NSG	/	27	Identify key mutations closely associated with the initiation of AML, especially *FLT3*-ITD *FLT3*-ITD mutation is highly enriched in LICs but is relatively less prevalent in stem cells capable of reconstituting multilineage hematopoiesis FLT3 pathway inhibition remains an effective strategy for eliminating AML cells despite the presence of diverse coexisting mutations
[31]	NSG, NSG-SGM3	73	8	While some subclones tend to engraft in PDX models preferentially, this trend does not always correlate with their frequency of appearance during disease relapse The proportion of subclones that successfully engraft is very low (< 10%) in the original samples, indicating that the engraftment potential of subclones is not solely determined by their abundance in the primary tumor Only a subset of subclones possesses significant engraftment potential Different mouse strains exert variable effects on the engraftment of specific subclones
[32]	NSG-SGM3	84	23	Trace clonal dynamics over 15 months using whole-exome sequencing and identify the expansion of certain cell clones that were undetectable at diagnosis Highlight the presence of multiple clones even at initial diagnosis and correlate clonal dynamics with genetic risk factors, suggesting that leukemias harboring numerous potentially expanding clones are more prone to chemoresistance and relapse Identify five clonal dynamics patterns: monoclonality, stability, loss, expansion, and blast emergence
[33]	NSG	2	2	Compared to primary recipients, successive recipient generations derived from the same donor exhibit a more similar clonal composition As continuous transplantation proceeds, clonal complexity significantly decreases, with some clones being lost during the transplantation process

PDX: Patient-derived xenograft; AML: acute myeloid leukemia; VAF: variant allele frequency; PT: primary tumor; FLT3: Fms-related tyrosine kinase 3; ITD: internal tandem duplication; LICs: leukemia-initiating cells.

The dynamic clonal landscape observed in the PDX models uncovers specific subgroups of acute leukemia cells. A small percentage of PDX ALL cells, referred to as leukemia-regenerating cells (LRCs), were resistant to chemotherapy but developed sensitivity without an *in vivo* environment^[34]^. Another study transduced B-ALL cells with microRNA-126 (miR-126) and identified the enrichment of overexpressing miR-126 cells under a four-week chemotherapy cycle of vincristine and dexamethasone^[35]^. PDXs also enlightened us with the relapse patterns, demonstrating that relapse could stem from preexisting subclones or newly acquired mutations during treatment^[36]^. Correspondingly, Shlush *et al.* uncovered that relapse originated from either rare LSCs found at diagnosis or larger subsets of immunophenotypically committed leukemia cells with strong stemness transcriptional signatures^[37]^. Specific genes have also been revealed in the clonal evolution of relapsed leukemia. For instance, ALL PDXs validated that the 5′-nucleotidase, cytosolic II (*NT5C2*) mutation was typically absent or present at very low frequencies at diagnosis. Still, they emerged as late events in the relapse process of ALL^[38]^.

Notably, the clonal dynamics of leukemia could be influenced by spatial heterogeneity and microenvironment. Thus, the clonal evolution might deviate from clinical circumstances^[39]^. However, more humanized PDXs are capable of alleviating clonal competition between leukemia subclones and retaining more clinically relevant variant allele frequencies (VAFs)^[19]^. Currently, achieving precision and consistency in mimicking the genetic characteristics of primary leukemia with PDXs remains a challenge that necessitates further exploration and refinement.

### Representation of the genetic landscape in the PDX model

Previous studies have reported that LSCs exhibited genetic diversity, mirrored subclonal patterns, and varied in their regenerative capacity^[40]^. An analysis of acute leukemia PDXs reported that 24 out of 48 pairs of PT-PDX (PT, primary tumor) samples exhibited somatic variant allele frequency (SVAF) changes less than 2-fold and SVAF > 10%^[30]^. Notably, low-VAFs were observed to be lost during the initial transplantation of ALL, but the PDX model exhibited high molecular stability after the initial xenotransplantation^[41]^.

Although most key mutations of acute leukemia like Fms-related tyrosine kinase 3 internal tandem duplication (*FLT3*-ITD) and nuclear pore protein 98-nuclear receptor-binding SET domain protein 1 fusion gene (*NUP98-NSD1*) were found to be reserved in PDXs^[42]^, the SVAFs of some key mutations like nucleophosmin 1 (*NPM1*), tumor protein p53 (*TP53*), and isocitrate dehydrogenase 2 (*IDH2*) showed greater divergence in PT-PDX pairs^[30]^, partly due to their roles at later stages of leukemogenesis and the mice-specific pressure on genetic evolution^[43]^. Furthermore, chromosomal instability in B-ALL PDXs is found to be highly relevant to primary samples^[44]^. When it comes to the differentially methylated genes, researchers identified that most hypermethylated genes remained transcriptionally silent in T-ALL PDXs^[45]^. In another study, the proportion of genes exclusively expressed in primary AML samples, as opposed to PDXs, only constituted a minor fraction of the total gene set, ranging from 1.6% to 5.1%^[42]^. In terms of protein abundance, an average of 57% of proteins showed equivalence (fold change < 1.5) between patient samples and their corresponding ALL PDXs^[46]^. In ALL PDXs, proteins related to cell cycle and proliferation exhibited significantly higher abundance^[46]^, while proteins related to immune response, cytokine production, and leukocyte activation were significantly lower^[45]^. In AML PDXs, there was a notable reduction in the expression of genes associated with immune system functioning, cell surface identification markers, transport mechanisms, and signal transmission processes^[42]^.

Therefore, further discussion is needed to determine whether the dominant subpopulations and evolution hierarchies in PDXs vary from those in human conditions, and how well PDXs can preserve the heterogeneity and genomic landscape of AML samples in novel PDXs.

### The supportive role of bone marrow microenvironment

The bone marrow microenvironment, commonly referred to as the bone marrow niche, encompasses two primary components: the endosteal niche and the perivascular niche. These niches are complex ecosystems comprising osteocytes, osteoblasts (OBs), osteoclasts, endothelial cells, diverse mesenchymal stem cells (MSCs), and many soluble factors^[47]^. They play a vital role in leukemia development and are intimately tied to disease resistance^[48,49]^, probably due to their influence on the clonal evolution of LSCs^[19,50]^. To enhance our understanding of the interactions between leukemia cells and the bone marrow microenvironment, PDXs have been widely used in numerous studies to simulate more humanized bone marrow conditions.

As previously discussed, key human factors present in mouse models, such as macrophage colony-stimulating factor (M-CSF), IL-3, GM-CSF, and thrombopoietin, significantly impact the engraftment of leukemia cells with various mutations by fostering the generation and self-renewal of LSCs^[50,51]^. In addition, modulating signaling factor receptors, as seen in CXC chemokine receptor 4 (CXCR4) and CD44, has demonstrated potential in downregulating markers that enhance LSCs’ self-renewal and treatment resistance^[52,53]^. The CXCR4 antagonist, plerixafor, was also found to induce the mobilization of ALL cells, thus enhancing the efficacy of cell cycle-specific drugs^[54]^. Aside from the intricate cross-talk between leukemia cells and their niche factors, researchers have been attracted to cellular interactions. *In vitro* drug screening using T-ALL PDX cells and endothelial cells is one of the promising methods to study leukemia-microenvironment interactions and identify therapeutic vulnerabilities^[55]^. Additionally, AML PDX femur analysis indicated that LSCs preferentially localized to the endosteal region of mouse bone marrow, an area rich in OBs^[16]^. Researchers also found that hindering the transfer of fatty acids from adipocytes (ADs) to AML cells impeded the progression of AML^[56]^. Conversely, increased ADs retarded the engraftment of leukemia in PDX models^[57]^. Furthermore, MSCs are indispensable in the *in vivo* development of AML cells and promote the migration of AML cells to the peripheral blood, spleen, and liver^[12]^.

### The protective role of bone marrow microenvironment

The bone marrow microenvironment could be the key to combating drug resistance. When faced with Ara-C, the bone marrow microenvironment provides the furin enzyme and further activates the transforming growth factor beta (TGF-β) signaling pathway, leading to drug resistance^[58]^. Additionally, under chemotherapy, levels of mouse mitochondrial DNA in AML cells increase, and LSCs that acquire mitochondria exhibit greater proliferation potential. Interestingly, in PDXs, stromal cells transfer mitochondria to leukemia cells^[59]^. Moreover, researchers found that the microenvironment may induce autophagy, resulting in drug resistance in PDXs, and targeting Bruton’s tyrosine kinase (BTK) overcame the resistance^[60]^. Similarly, combined panobinostat (histone deacetylase inhibitor) with chemotherapy counteracts the supportive interactions between leukemia cells, bone marrow stromal cells, and the extracellular matrix (ECM)^[61]^.

Conversely, leukemia cells can influence the bone marrow microenvironment and may shape a conducive niche that benefits their survival and proliferation. The AML engraftment has been found to contribute to functional and structural abnormalities of the bone marrow vasculature in PDXs, including loss of sinusoidal structures, reduction in vessel diameter, poor perfusion, and hypoxia. This engraftment also leads to increased vascular permeability, which may be attributed to the over-activation of nitric oxide synthase 3 (NOS3), ultimately elevating nitric oxide (NO) levels in the bone marrow^[62]^. Additionally, PDX models engrafted with *FLT3*-ITD+ AML cells exhibit changes in vascular morphology, with a decrease in CD31+Sca-1^high^ (Sca-1, stem cell antigen-1) endothelial cells and an increase in CD31+Sca-1^low^ endothelial cells^[63]^. Furthermore, AML-secreted kynurenine binds osteoblastic serotonin receptor 1B (HTR1B) to induce proinflammatory serum amyloid A (SAA), which in turn upregulates indoleamine 2,3-dioxygenase 1 (IDO1) in leukemia cells to amplify kynurenine production and establish a self-reinforcing loop, which fosters immune evasion^[64]^. Other secretions, like exosomes, have also been shown to impair bone marrow hematopoiesis and mobilize hematopoietic stem and progenitor cells (HSPCs) to the peripheral blood. In PDX models, the AML exosomes downregulate retention factors in stromal cells and directly inhibit HSPC function^[65]^. Moreover, the RNA and protein cargo within AML exosomes regulate multiple hematopoietic transcription factors and stromal cell regulatory genes, ultimately remodeling the bone marrow microenvironment to favor leukemia progression^[65]^. In ALL PDXs, large extracellular vesicles are also observed to be secreted by leukemia cells and subsequently absorbed by MSCs^[66]^, which eventually leads to impaired bone marrow hematopoiesis^[67]^.

### Innovative ways to model bone marrow microenvironment

To better simulate the interaction of patient-derived cells and bone marrow microenvironment, researchers have embarked on innovative designs, deviating from conventional PDXs. There is an *in vitro* culture system featuring immortalized MSCs, a hypoxic (3% O_2_) environment with plasma-like amino acid and cytokine concentrations. This model allows for a precise assessment of the effects of ruxolitinib and chemotherapy on G protein-coupled receptor 56-positive (GPR56+) LSCs and differentiating cells^[68]^. In a different direction, a humanized bone marrow microenvironment was reconstructed within an ossicle^[19]^, while another study adopted a biomimetic 3D structure composed of engineered hydroxyapatite and type I collagen, co-transplanting MSCs, OBs, endothelial cells, and inflammatory factors^[69]^. For enhanced drug screening efficiency, the zebrafish model provides a more practical choice for high-throughput studies, despite limitations in the reconstitution of the leukemia microenvironment in the zebrafish model^[70,71]^.

## CURRENT EFFECTIVENESS OF ACUTE LEUKEMIA PDX IN STUDYING RESISTANCE

### The quality of existing PDXs

The evolving technology of engraftment and further refinements have dramatically improved the utility of acute leukemia PDXs as co-clinical or preclinical platforms. Nevertheless, given the stark differences in the bone marrow microenvironment between human and mouse models, a key question arises: To what extent can PDX models accurately predict clinical outcomes for acute leukemia patients? Previously, we summarized the biological characteristics of leukemia cells in PDXs, and the results showed that PDX can support the exploration of drug resistance mechanisms in multiple aspects. In solid tumors, numerous studies have demonstrated the co-clinical potential of PDX models by comparing drug responses between PDXs and their respective patient cohorts, yielding promising results^[72-74]^. As previously concluded, studies have demonstrated that well-established PDX models successfully retain clinical samples’ major pathological, phenotypic, and genetic signatures, mirroring the drug sensitivity profiles observed in patients^[75]^. For instance, a co-clinical study of mivebresib, a bromodomain and extra-terminal (BET) inhibitor, in AML demonstrated that PDX response significantly correlated with clinical activity in terms of leukemia blast reduction. However, the association between bone marrow and spleen responses in PDXs and clinical outcomes was less evident^[76]^. In addition, in pediatric AML PDXs, certain genetic subtypes of pediatric AML exhibit challenges in primary engraftment and serial transplantation^[77]^.

It should be noted that a wider range of samples and more replicates are expected to be applied in future studies, as well as cutting-edge technologies to ensure engraftment rates and PDX quality. Nowadays, the PDX models have emerged as a shared resource for the global cancer research community, serving as a tremendous platform for biomarker discovery and drug screening. When combined with massive data analysis like machine learning, the multi-omics datasets of PDXs lay the foundation for the prediction of clinical response^[78]^. Furthermore, these comprehensive databases provide instrumental references for devising effective treatment strategies and tailoring therapies to individual patients^[73,79]^. The consistent alterations in PDX cohorts of different samples also indicated underlying confounding in the experiment results^[42]^. For acute leukemia, the Public Repository of Xenografts (PRoXe), a large PDXs biobank, demonstrates the potential of performing human-like clinical experiments using PDXs^[80]^, which also provides convenient access to PDX cells for experiments. Recently, another ALL biobank established in Japan claimed great consistency with primary samples and achieved an engraftment rate of 93.3%^[81]^.

### Novel technology and application

The use of novel technologies has extensively broadened the application of PDXs, usually combining genomic editing and imaging technologies. For instance, researchers created an *in vivo* screening platform with CRISPR-CAS9 and PDXs to discover and validate potential targets^[82-84]^. RNA interference technology is crucial in studying the function of certain genes. For instance, one study adopted inducible RNA interference to study the silence of the *MCL1* gene and transduced mCherry fluorescent protein and Gaussia luciferase for *in vivo* imaging^[85]^. Moreover, the integrated multi-omic profiling is critical for the identification and validation of potential pathways in acute leukemia PDXs, apart from mice survival evaluation and leukemia burden analysis^[86]^. Notably, apart from PDX, the mouse model is also preferred by other stem cell studies, including key mutation knock-in in HSCs^[87]^, reconstitution of a functional immune system^[9]^, and *in vivo* evaluation of induced pluripotent stem cell-derived HSCs (iHSCs)^[88]^.

Alternative models exhibit distinct advantages that render them preferentially applicable in specific studies. CDXs, immunodeficient mice engrafted with cell lines, offer enhanced convenience and availability for standardized experimentation. GEM models recapitulate spontaneous tumorigenesis through targeted genetic modifications, providing valuable platforms to investigate cancer genetics and therapeutic resistance mechanisms. Zebrafish models, owing to their optical transparency, rapid development, and genetic tractability, are ideal for high-throughput drug screening and real-time imaging of cancer metastasis or angiogenesis. Notably, novel *ex vivo* models are promising to interrogate leukemia microenvironmental interactions. Organ-on-a-chip technologies incorporate microfluidic architectures with advanced biosensing capabilities to simulate critical features of hematopoietic niches^[89]^; 3D *in vitro* models utilize biomaterials to model ECM and provide a 3D environment^[90]^. The selection of the optimal model necessitates meticulous consideration of the research objectives and available resources, as each model exhibits unique strengths and limitations (summarized in Table 2).

**Table 2:** Emerging acute leukemia models compared with the PDX model

**Feature**	**Conventional PDX**	**CDX**	**GEM**	***Ex vivo* (On-chip)**	***Ex vivo* (3D structure)**	**Zebrafish**
Time economy	Low	Low	Low	High	Medium	High
Cost affordability	No	No	No	Yes	Yes	Yes
Design flexibility	Low	Medium	Medium	High	Medium	Medium
High-throughput screening	Low	Low	Low	High	Medium	Medium
Direct observation of HSC	No	No	No	Yes	Possibly	Yes
Extramedullary involvement	Yes	Yes	Yes	No	No	Possibly
Bone marrow interaction	High	High	High	Medium	Medium	Low
Bone marrow changes	High	High	High	Medium	Medium	Low
Clinical consistency	High	Medium	Medium	Low	Medium	Medium

PDX: Patient-derived xenograft; CDX: cell-line-derived xenograft; GEM: genetic engineered mouse; On-chip: lab-on-a-chip; HSC: hematopoietic stem cell.

### Current challenges for PDXs

Carefully handling factors, such as stromal cell composition and the status of the immune system, that pose limitations to PDX models remains an obstacle. Clinical realities like spatial heterogeneity, comorbidity management, pharmacokinetic disparities, dietary factors, and time constraints complicate the faithful replication of clinical conditions in PDX systems^[6]^. Additionally, due to the incomplete mimicry of human acute leukemia conditions, the heterogeneity and clonal architecture of leukemia potentially differ from the primary samples, especially after serial passages. As preclinical models, acute leukemia PDXs are perfect for exploring evolution patterns and studying specific dominant clones that persist over passages or under drug pressure; when serving as co-clinical models, the PDXs are expected to faithfully retain the primary heterogeneity through limited passages, providing immediate insights into agent sensitivity^[6]^.

## THERAPY RESISTANCE IN ACUTE LEUKEMIA PDXS

Despite differences in microenvironment components, clonal evolution, and signaling pathways, the PDX model remains a robust humanized preclinical model of acute leukemia due to its preservation of major histological and genetic features and phenotypes of donor tumors and consistency in the passages^[5]^. Moreover, the PDX model has a similar response to treatment as acute leukemia patients and thus is optimal for the preclinical evaluation of the efficacy of therapeutic approaches and their effect on normal hematopoiesis^[91]^. In this section, we present you with the latest application of PDXs in evaluating therapeutic efficacy.

### Acute leukemia PDX model in targeting mutated proteins

#### Combating drug resistance

PDX models of AML and ALL with various mutations are instrumental in elucidating resistance mechanisms to mutated protein inhibitors and investigating strategies to combat drug resistance. Zhang *et al.*, for instance, discovered that a multikinase inhibitor targeting both FLT3 and BTK effectively overcame FLT3 inhibitor resistance in AML PDXs, which was mediated by autophagy^[60]^. Liu *et al.* targeted signal transducer and activator of transcription 5 (STAT5) signaling to suppress stemness genes and overcome resistance to isocitrate dehydrogenase (IDH) inhibitors in AML PDXs^[92]^. Additionally, Hegde *et al.* aimed at the VAV guanine nucleotide exchange factor 3/ras-related C3 botulinum toxin substrate (VAV3/RAC) pathway in B-ALL PDXs and combated tyrosine kinase inhibitor (TKI) resistance induced by breakpoint cluster region-Abelson murine leukemia viral oncogene homolog 1 (*BCR-ABL1*) transformation^[93]^.

With the advancement in sequencing technologies and molecular structure, the genetic landscape of acute leukemia has been unraveled, revealing a plethora of oncogenic mutations and brand-new docking sites that can be exploited as resistance targets. In one study, the novel mutant IDH2 inhibitor SH1573 exhibited better therapeutic effect than the older generation of mutant IDH2 inhibitors, partially due to the novel binding site (R140Q) on mutant IDH2^[94]^. Similarly, different TKIs demonstrated varied therapeutic effects on the *FLT3* mutation; for example, the *FLT3*-ITD-F691L mutation confers resistance to gilteritinib, but sitravatinib does not rely on the F691 residue^[95]^.

#### Exploring drug combinations

In recent studies leveraging acute leukemia PDX models, researchers have illuminated the potential of combined therapeutic strategies targeting multiple mutated proteins, notably FLT3 and associated pathways. Janssen *et al.* reported a notable synergy between venetoclax and gilteritinib in *FLT3* wild-type high-risk AML, mediated through the suppression of the antiapoptotic protein myeloid cell leukemia-1 (Mcl-1)^[96]^. Analogously, Li *et al.* observed compelling cooperation between Notch and FLT3 inhibitors in *FLT3*-ITD+ AML, which selectively reduced cellular proliferation and promoted apoptosis^[97]^. Moreover, Long *et al.* demonstrated that histone deacetylase 8 (HDAC8) inhibition reactivates cellular tumor antigen p53 (p53), thereby overcoming TKI resistance in *FLT3*-ITD+ AML PDX models. This resistance was induced by HDAC8 upregulation, which inactivates p53^[98]^. Saito *et al.* adopted a combination of FLT3 TKI and B-cell lymphoma 2 (Bcl-2) inhibitor in AML PDXs and identified prominently higher sensitivity to apoptosis induction compared to monotherapy^[18]^. Notably, DNA methyltransferase inhibitors (DNMTis), such as azacitidine, exhibit marvelous enhancement of other targeted therapies, including FLT3, IDH1, and IDH2 inhibitors^[5]^. Consequently, the synergistic effects observed with DNMTis are frequently explored in targeting mutated proteins.

### Acute leukemia PDX model in targeting apoptosis

#### Uncovering drug resistance mechanisms

PDX models replicate the drug response observed in patients and offer insights into mechanisms underlying drug resistance against apoptosis. In one study, PDXs were used to represent distinct therapeutic outcomes of venetoclax in AML subsets with *NPM1/RAD21* (RAD21 cohesin complex component) *vs. TP53/TET2* (ten-eleven translocation 2) mutations^[99]^. To identify potential targets in AraC resistance AML, Larrue *et al.* uncovered that the proportion of CALCRL+ cells increased after AraC treatment in PDXs^[25]^. Correspondingly, Farge *et al.* developed chemoresistant AML PDXs and discovered that AraC treatment was followed by elevated reactive oxygen species and active mitochondria, which suggested a potential strategy involving combined targeting of mitochondrial metabolism^[100]^. Moreover, in the Philadelphia chromosome-like B-cell acute lymphoblastic leukemia (Ph-like ALL) PDXs treated with ruxolitinib, the myelocytomatosis oncogene (*MYC*) was suppressed at first, but recovered its expression after long-term treatment and presented as the turning point for the expansion of resistant cells^[101]^. In another study, researchers adopted CRISPR/CAS9 screens in ALL PDXs and identified broad dependency on Bcl-2, BRCA1-interacting protein C-terminal helicase 1 (BRIP1), and COP9 signalosome subunit 2 (COSP2)^[84]^.

#### Overcoming glucocorticoid resistance

Glucocorticoids (GCs) are an indispensable part of the ALL treatment. However, the GC resistance has not been fully elucidated and remains an issue for doctors and researchers. In one study, six T-ALL PDXs were sensitive to GC, but five of them relapsed and developed dexamethasone resistance. Notably, the resistance-related biological changes were found to be highly individualized^[102]^. These findings highlight the necessity for precision medicine, and the PDX model is a good choice for resistance investigation. Beck *et al.* conducted a study comparing GC-sensitive and GC-resistant ALL PDX models. They found that the transcription factor PU.1 blocks GC receptor binding by occupying chromatin regions^[103]^. Comparatively, in the mixed lineage leukemia-rearranged (*MLL*-r) B-ALL PDXs, neuron-glial antigen 2 (NG2) suppresses the expression of nuclear receptor subfamily 3 group C member 1 (*NR3C1*) that encodes the GC receptor, and this suppression occurs through the *FLT3*/AP-1 (activator protein 1) signaling axis^[104]^.

The GC resistance can also be induced by Bcl-2 overexpression. Elevated Bcl-2 expression neutralizes the pro-apoptotic effect of GC in ALL PDXs, and targeting the Bcl-2 signaling has demonstrated therapeutic effects in various types of ALL PDXs. One study reported that dexamethasone increases IL-7Rα expression and strengthens IL-7/JAK/STAT5 (JAK, Janus kinase) signaling activity, subsequently elevating Bcl-2 protein levels. Using the T-ALL PDXs, researchers successfully reversed GC-induced resistance using the JAK inhibitor (ruxolitinib)^[105]^. In another early T-cell precursor ALL (ETP-ALL) PDX experiment, pimozide inhibited the activation of STAT5 and increased apoptosis sensitivity^[106]^. Direct inhibition of Bcl-2 using venetoclax also overcame the GC resistance in *MLL*-ALL PDXs^[107]^. Moreover, GC resistance can be reverted by inhibiting the pro-survival signaling. For example, in the T-ALL PDXs, dasatinib re-sensitized dexamethasone resistance through lymphocyte cell-specific kinase (LCK) inhibition^[108]^. Laukkanen *et al.* identified a promising strategy combining LCK and mammalian target of rapamycin complex 1 (mTORC1) targeting, which effectively counteracted compensatory survival mechanisms^[109]^.

#### Evaluating novel combinations

Solely targeting apoptosis often performs poorly due to drug resistance in AML, whereas combining it with other targeted drugs tends to enhance their efficacy significantly. For instance, studies have shown that caloric restriction (CR) is effective, but the non-genetic selection of LSC will lead to the recurrence of the condition. To address this challenge, Pallavi *et al.* developed LSD inhibitors and validated their effectiveness in combination with CR using PDX models^[110]^. Targeting cytosolic and mitochondrial proteins that prevent cell death, such as Bcl-2, Mcl-1, X-linked inhibitor of apoptosis protein (XIAP), and Aurora kinase B (AURKB), is one of the important ways to combat apoptosis resistance, as evidenced in AML and ALL PDXs^[111,112]^.

One of the primary objectives of combined therapies is to target Bcl-2 resistance, mainly by inhibiting Mcl-1. The overexpression of Mcl-1 promotes drug resistance by regulating metabolism (the tricarboxylic acid cycle, glycolysis) and the interaction between leukemia cells and the stroma. The combined inhibition of Mcl-1 and Bcl-2 can release a series of pro-apoptotic proteins^[113]^. Ramsey *et al.* introduced VU661013, a highly selective Mcl-1 inhibitor, demonstrating its efficacy in venetoclax-resistant AML cells and PDX models derived from Mcl-1-dependent patients^[114]^. Similarly, Bhatt *et al.* reported a shift in Bcl-2 interacting mediator of cell death (BIM) binding from Bcl-2 to Mcl-1 in venetoclax-resistant AML PDXs, emphasizing the need for concurrent inhibition of both Bcl-2 and Mcl-1 for optimal efficacy^[115]^. Moreover, Lewis *et al.* induced ceramide accumulation and subsequent Mcl-1 degradation in PDX models, effectively overcoming venetoclax resistance^[116]^. Similarly, usnic acid has been proven effective in restoring venetoclax sensitivity in AML PDXs via Mcl-1 degradation^[117]^. In ALL PDXs, reconstitution of BIM-mediated regulation over antiapoptotic Bcl-2 family proteins can also confer GC resistance^[118]^.

Additionally, Tahir *et al.* investigated the synergistic effect of combining venetoclax with eftozanermin, which activated the death receptor 4/5, thereby inducing apoptosis through the extrinsic pathway^[119]^. Additional pathways found to enhance the efficacy of venetoclax in PDXs include fatty acid oxidation inhibition^[120]^, protein phosphatase 2A activation (PP2A)^[121]^, and peroxisome proliferator-activated receptor alpha (PPARα) stimulation^[122]^. Interestingly, sequential administration can be used as a combination strategy to improve the therapeutic effect in PDXs. Specifically, pretreatment with JQ-1 induced upregulation of *MYC* in AML cells, subsequently sensitizing these cells to venetoclax, which effectively triggered an evolutionary trap against drug resistance^[123]^. These studies underscore the potential of PDX models in capturing the intricate heterogeneity present in leukemia cells exhibiting drug resistance, thereby addressing the necessity to evaluate the effectiveness of novel drug options within their respective genomic contexts.

### Acute leukemia PDX model in epigenetic targets

PDXs exhibit a notable capacity to replicate the epigenetic characteristics of clinical leukemia samples and are eligible for related studies. Researchers have applied RNA interference, CRISPR/Cas9 gene editing, plasmid transfection, and lentiviral transduction techniques to PDXs to investigate epigenetic modulators. In a study employing shRNA, researchers silenced YTH domain-containing protein 1 (*YTHDC1*) and observed a marked decrease in AML engraftment and a significant delay in leukemogenesis^[124]^. In a parallel vein, another study knocked down zinc finger protein 217 (*ZNF217*) in B-ALL PDXs and found that leukemia progression was prominently impeded^[125]^. The identification of epigenetic targets indicates their great potential in overcoming leukemia resistance through histone modification, DNA methylation, and non-coding mRNA.

Although the DNMTis have demonstrated marvelous potential in AML, many epigenetic therapies remain to be improved and evaluated in preclinical models before entering clinical practice. For example, the disruptor of telomeric silencing 1-like (DOT1L) is tightly related to the *MLL*-r and promotes the expression of downstream genes. However, solely targeting DOT1L yielded adaptive resistance^[126]^. This indicates the need for novel targets, sequential designs, and drug combinations to improve the potential of epigenetic targets. The DNMTi 5-azacytidine has been proven to be effective in AML and exhibited robust therapeutic efficacy when combined with various therapies. Notably, ALL with specific mutations can also benefit from TKIs, making it a powerful candidate for overcoming drug resistance^[127]^. More instances and comprehensive details are outlined in the accompanying Table 3, emphasizing the invaluable role of PDX models in unraveling epigenetic mechanisms and expediting the development of targeted acute leukemia therapies.

**Table 3:** Epigenetic therapies tested in the PDX model

**Ref.**	**Target**	**Drug name**	**Leukemia**	**Key observations**
[128]	METTL3	STM2457	AML	STM2457 directly binds to the METTL3-METTL14 heterodimer, competitively inhibiting the binding site of SAM, thus blocking METTL3’s catalytic activity. This provides a distinct therapeutic window for drug-resistant leukemia
[129]	METTL3	GRh2	APL	Lactylation-driven METTL3 overexpression promotes all-trans retinoic acid resistance, and GRh2 restores sensitivity by inhibiting lactylation and METTL3
[130]	SENP1	Momordin-Ic	AML	SIRT3-mediated desumoylation activates fatty acid oxidation by inhibiting HES1, and SENP1 inhibition reverses chemotherapy resistance
[131]	MDM2	Nutlin3A	AML	*MTF2* deficiency leads to MDM2 upregulation and p53 degradation, thereby inhibiting apoptosis and DNA damage repair. MDM2 inhibitors overcome chemoresistance induced by MTF2 deficiency
[132]	PRC2	GSK126	ALL	*NSD2* mutation leads to the accumulation of H3K27me3 in the promoter region of GR, inhibiting the expression of GR. The PRC2 inhibitor can remove the H3K27me3 marker in the promoter region of GR, restoring the expression of GR
[86]	PRC2	EPZ-6438	AML	EPZ-6438 treatment inhibits the activity of the catalytic subunit EZH2 within PRC2, thereby preventing trimethylation of H3K27me3, which, in turn, upregulates the expression of HLA class II and prevents immune escape
[133]	BET	JQ1	T-ALL	BET inhibitor, JQ-1, enhances the efficacy of venetoclax by upregulating the pro-apoptotic protein BIM and downregulating the antiapoptotic protein Bcl-2
[52]	BET	ARV-825	AML	As compared with traditional BRD4 inhibitors, ARV-825 can downregulate BRD4 and related transcriptional targets more durably, thereby overcoming the resistance to traditional BRD4 inhibitors
[134]	LDH	Oxamate	AML	AML can bypass the inhibition of glycolysis by BET inhibitors by using lactic acid as an alternative carbon source, and maintain mitochondrial function and cell survival. Inhibiting the utilization of lactic acid can block this metabolic escape pathway
	BET	INCB054329		
[135]	BET	JQ1	AML	AMPK partially restores histone acetylation by maintaining a certain level of acetyl-CoA, thereby attenuating JQ1’s anticancer effect. When used in combination, Compound C further reduces acetyl-CoA levels and histone acetylation by inhibiting AMPK, overcoming JQ-1 resistance
	AMPK	Compound C		
[136]	LSD1	GSK2879552	ETP-ALL	Inhibition of LSD1 increases the level of H3K4me2 in the promoter or enhancer region of the *BIM* gene, leading to an increase in BIM expression and neutralizing the antiapoptotic effect of Bcl-2
[137]	CDK4/6	Palbociclib	BCP-ALL	When the expression of miR-497/195 is low, the CDK4/6 pathway is activated, and cell proliferation is accelerated. Palbociclib targets CDK4/6 to overcome miR-497/195-mediated chemotherapy resistance
[138]	YTHDC1	EPZ-5676	B-ALL	YTHDC1 inhibition affects DNA damage repair by regulating the KMT2C-H3K4me1/me3 axis, and increases the sensitivity of B-ALL cells to chemotherapy
[139]	FACT complex	CBL0137	ALL	CBL0137 traps the FACT complex in the chromatin through chromatin-trapping. This inhibits the FACT-mediated DNA repair and increases the sensitivity of B-ALL cells to chemotherapeutic drugs
[140]	HDAC3	RGFP966	APL	HDAC3i increases the acetylation level of the PML-RARα protein and promotes its SUMOylation and ubiquitination. This makes the PML-RARα protein more easily recognized and processed by the degradation pathway induced by ATRA

PDX: Patient-derived xenograft; METTL3: methyltransferase-like 3; AML: acute myeloid leukemia; SAM: S-adenosylmethionine; APL: acute promyelocytic leukemia; SENP1: sentrin-specific peptidase 1; SIRT3: sirtuin 3; HES1: hairy and enhancer of split 1; MDM2: murine double minute 2; MTF2: mixed lineage leukemia 2, histone-lysine N-methyltransferase; p53: tumor protein p53; PRC2: polycomb repressive complex 2; ALL: acute lymphoblastic leukemia; NSD2: nuclear receptor binding SET domain protein 2; GR: glucocorticoid receptor; H3K27me3: histone H3 lysine 27 trimethylation; EZH2: enhancer of zeste homolog 2; HLA class II: major histocompatibility complex class II; BET: bromodomain and extra-terminal domain; T-ALL: T-cell acute lymphoblastic leukemia; BIM: BCL-2 interacting mediator of cell death; BcL-2: B-cell lymphoma 2; BRD4: bromodomain-containing protein 4; LDH: lactate dehydrogenase; AMPK: adenosine monophosphate-activated protein kinase; LSD1: lysine-specific demethylase 1; ETP-ALL: early T-cell precursor acute lymphoblastic leukemia; CDK4/6: cyclin-dependent kinase 4/6; BCP-ALL: B-cell precursor acute lymphoblastic leukemia; miR-497/195: microRNA-497/195; YTHDC1: YTH domain-containing protein 1; B-ALL: B-cell acute lymphoblastic leukemia; KMT2C: lysine methyltransferase 2C; H3K4me1/me3: histone H3 lysine 4 monomethylation/trimethylation; FACT: facilitates chromatin transcription; HDAC3: histone deacetylase 3; HDAC3i: histone deacetylase 3 inhibitor; PML-RARα: promyelocytic leukemia-retinoic acid receptor alpha; SUMOylation: small ubiquitin-like modifierylation; ATRA: all-trans retinoic acid.

### Acute leukemia PDX model in immunotherapy research

#### Uncovering immune interactions and assessing therapy safety through PDXs

The absence of a functional immune system is one of the obstacles that limit the use of PDX models in immunotherapy research. Recent advancements have seen the successful reconstitution of a functional hematopoietic system in PDX models by engrafting human HSPCs^[9,141,142]^. For instance, the MISTRG mice, developed with humanized IL-6, demonstrate effective hematopoiesis within 10-12 weeks of HSPC implantation. This model reveals the presence of activated and exhausted CD8+ T cells within the tumor microenvironment, marked by the expression of genes like *CD69*, *STAT1*, and *CXCR4*, highlighting its potential in unraveling immune-AML dynamics^[143]^. Furthermore, to investigate the effects on HSPCs and immune cells, healthy HSPCs or relevant functional immune cells can be injected into PDXs.

#### Novel drug evaluation

Existing immunotherapies have significantly advanced acute leukemia treatment. This includes immune checkpoint inhibitors (ICIs), immune cell engagers (ICEs), antibody-drug conjugates (ADCs), chimeric antigen receptor (CAR) T therapies, and associated signaling pathways. However, their efficacy is limited to a small fraction of patients due to the scarcity of effective targets and adaptive resistance. To broaden the reach and effectiveness of immunotherapies, there is an urgent need for innovative strategies and preclinical evaluation.

ICIs have emerged as one of the most promising immunotherapies in recent years. In one study, the human-mouse chimeric version of T-1A5 (ChT-1A5) inhibited the immunosuppressive function of B7 homolog 3 protein (B7-H3) and induced specific antibody-dependent cell-mediated cytotoxicity (ADCC) exclusively toward B7-H3+ AML cells in PDXs^[144]^. Additionally, AML cells can be sensitized to ICIs. The short 5′-triphosphate-modified RNA activates cytoplasmic immune receptor retinoic acid-inducible gene-I (RIG-I), increasing programmed death ligand-1 expression on AML cells. This establishes therapeutic sensitivity to anti-programmed cell death protein-1 checkpoint blockade^[145]^.

Moreover, leukemia-specific antibodies targeting cell surface antigens are capable of activating immune cells in PDX models. For instance, the *in vivo* infusion of NK cells, combined with CD33 monoclonal antibody, effectively reduced tumor burden in AML PDXs^[146]^. Similarly, another study employed the humanized CD200 antibody (IgG1) to block the immunosuppressive interaction with the CD200 receptor while eliciting a potent Fc-mediated immune response^[147]^. Furthermore, agents that upregulate the expression of immune-related surface antigens on leukemia cells may enhance the immune response. This involves regulating the expression of human leukocyte antigen class II (HLA-II)^[86]^, major histocompatibility complex class II (MHC-II)^[148]^, and CD123^[149]^. Additionally, the upregulation of immune antigens on leukemia cells can be induced by the secretion of inflammatory cytokines. A study uncovered that T cell therapeutic effects were improved through local release of interferon gamma (IFN-γ) and subsequent upregulation of MHC-II expression^[150]^.

ICEs connect antigens on leukemia cells to immune cell receptors. They allow for targeted immune activation, assuring leukemia eradication while avoiding off-target effects and collateral damage common with standard antibody therapy. ICEs targeting alternative targets are valuable in overcoming resistance caused by antigen loss and genetic heterogeneity^[151]^. Interestingly, for AML treatment, TKI can increase the mature glycosylated form of FLT3 and reposition the *FLT3*-ITD and *FLT3*-D835Y mutants, which are originally located in the endoplasmic reticulum, to the cell membrane. This cellular repositioning creates an ideal target landscape for immunotherapeutic interventions. Consequently, FLT3-targeted immunotherapies, such as FLT3/CD3 bispecific antibodies, can exert enhanced therapeutic effects^[152]^. However, for targeted therapy, especially immunotherapy, a higher surface marker level does not necessarily relate to a better therapeutic effect. In one study, some CD123+ ALL PDXs were resistant to the ADC drug PVEK but exhibited vulnerability to PVEK’s cytotoxic payload, indicating that the antibody portion targeting CD123 may fail to be internalized in the resistant leukemia cells^[153]^.

#### Improving CAR-T therapy

CAR-T therapy has demonstrated remarkable clinical efficacy in recent years^[154]^. However, certain factors may contribute to the resistance and relapse events continuously popping up, such as antigen loss^[155]^, the Galectin-9-T-cell immunoglobulin and mucin-domain containing-3 (LGALS9-TIM3) immune checkpoint axis^[156]^, cell-intrinsic death receptor signaling impairment^[157]^, and the immunosuppressive microenvironment^[158]^. Thus, it is crucial to perform preclinical experiments for further investigations. Mandal *et al.* adopted structural surfaceomics techniques and identified the activated conformation of integrin beta2 (aITGB2) as the AML-specific CAR-T target to provide an alternative target for treatment-resistant AML^[159]^. Additionally, combined therapy is important in overcoming CAR-T resistance. For instance, the 5′-azacitidine (AZA) treatment has been found to elevate surface marker levels and improve CAR-T targeting efficacy in AML PDXs^[149]^. To prevent tumor escape and resistance caused by antigen loss, researchers constructed CAR-T cells targeting more antigens, such as CD19 and CD123. The co-targeting of CD19 and CD123 exhibited notable resistance to antigen escape in ALL PDXs, even when CD19 expression was low^[160]^.

As for alleviating the side effects of CAR-T therapy, researchers have tested various innovative approaches using PDXs, including the use of epitope-edited CAR-T-resistant HSPCs^[161]^, the development of engager-dependent T cells^[162,163]^, and the identification of targets that facilitate CAR-T elimination^[164,165]^. Furthermore, in order to prevent resistance caused by fratricide, researchers employed CRISPR/Cas9 technology to knock out the *CD7* gene in CAR-T cells^[166]^. Interestingly, an alternative CAR-T design confers fratricide resistance by expressing an anti-CD7 protein expression blocker (PEBL) and demonstrates marvelous clinical efficacy^[167]^. More potential immunotherapies validated in PDX models are summarized in Table 4.

**Table 4:** Improving immunotherapy in the PDX model

**Ref.**	**Category**	**Drug name**	**Target**	**Subject**
[168]	ICI	Pembrolizumab	PD-1	Pembrolizumab inhibits PD-1 and improves the therapeutic effect of blinatumomab (ICE). Concurrent administration of pembrolizumab overcomes immune checkpoint resistance and effectively eliminates MRD in PDXs
[169]	ADC	Anetumab ravtansine	MSLN	Anetumab ravtansine binds specifically to cells expressing MSLN, subsequently delivering the cytotoxic agent DM4 into these cells, ultimately inducing cellular apoptosis
[170]	ADC	20D9-ADC	FLT3	The 20D9-ADC employs P5 conjugation technology, achieving stable antibody conjugation via ethynylphosphoramidate. It boasts a high drug-to-antibody ratio and utilizes MMAF as its payload. And its ability to bind to Fcγ receptors, such as CD64, through its IgG1 Fc region, enhances therapeutic efficacy
[171]	Antibody	Teplizumab, OKT3	CD3	Under CD3 antibody treatment, the TNFR1/NF-κB signaling cascade is activated, thus disrupting TCR-induced leukocyte apoptosis. The SMAC mimetic can selectively inhibit cIAP1 and cIAP2 proteins. This inhibition abrogates the NF-κB-dependent pro-survival axis within the TNF signaling pathway, redirecting cellular fate toward RIPK1-dependent apoptotic pathways and overcoming resistance
[172]	Antibody	OMP-52M51	NOTCH1	Anti-NOTCH1 monoclonal antibody downregulates metabolic pathways, particularly those involved in purine metabolism. This metabolic reprogramming renders leukemia cells more susceptible to antimetabolite chemotherapeutics
		Hu5F9-IgG2s	CD47	
[173]	CART	CD1a-CART	CD1a	Compared with CD1a-CAR T cells, CD1a-STAb T cells secrete CD1a/CD3 T cell engagers to recruit bystander T cells, enabling them to exhibit superior efficacy over CD1a-CAR T cells at lower effector-to-target ratios
[174]	CART	TSLPRCART	TSLPR	Ruxolitinib inhibits the JAK/STAT signaling pathway, blocking aberrant proliferation and survival signals induced by *CRLF2* rearrangement. This overcomes the TSLPRCART resistance in ALL PDXs
[175]	CART	H24 nanoCARs	CD72	Humanize the framework regions of the original llama-derived nanobodies and screen out clones with better anti-tumor abilities than the parental NbD4 nanoCAR. The H24 nanoCARs have been found to effectively target ALL relapses after CD19 CAR-T therapy
[176]	CART	CD19 CART	CD19	Azacitidine upregulates *TNFSF4* gene expression in leukemia cells, increasing OX40L production. OX40L binds to OX40 receptors on CAR T cells, activating costimulatory signals that enhance CAR T cell proliferation, effector differentiation, and long-term survival
[177]	CART	MSLN CART	MSLN	MSLN is expressed in AML cells but not in normal HSCs and progenitor cells, providing an alternative therapeutic target for resistant leukemia
[178]	CART	B7-H3.CAR- 28-T	B7-H3	B7-H3.CAR-28-T specifically recognizes and binds to the B7-H3 protein expressed on the surface of AML cells, providing an alternative therapeutic target for resistant leukemia
[179]	CART	AdCART	N/A	AMs, such as LLE-aCD33 and LLE-aCD38, redirect AdCAR-T cells to specific AML-related antigens. Adjusting the ratios of AMs can more precisely target drug-resistant cells of particular subtypes
[180]	CAR-NK	CAR.CD123-NK	CD123	The CAR.CD123-NK exhibits remarkable efficacy and better safety than CAR.CD123-T cells, opening an innovative avenue for resistant AML
[181]	CAR-NK	FLT3CAR_sIL-15NK	FLT3	FLT3CAR_sIL-15NK secretes sIL-15, which enhances the *in vivo* persistence of NK cells and activates T cells. This improves its therapeutic efficacy on resistant leukemia cells

PDX: Patient-derived xenograft; ICI: immune checkpoint inhibitor; PD-1: programmed cell death protein 1; ICE: immune checkpoint inhibitor; MRD: minimal residual disease; PDXs: patient-derived xenografts; ADC: antibody-drug conjugate; MSLN: mesothelin; DM4: N2′-deacetyl-N2′-(3-mercapto-1-oxopropyl)-maytansine; FLT3: FMS-like tyrosine kinase 3; MMAF: monomethyl auristatin F; Fcγ: fragment crystallizable gamma; IgG1: immunoglobulin G1; CD: cluster of differentiation; TNFR1: tumor necrosis factor receptor 1; NF-κB: nuclear factor kappa-light-chain-enhancer of activated B cells; TCR: T cell receptor; SMAC: second mitochondria-derived activator of caspases; cIAP1/2: cellular inhibitor of apoptosis protein 1; cIAP2: cellular inhibitor of apoptosis protein 2; RIPK1: receptor-interacting serine/threonine-protein kinase 1; NOTCH1: NOTCH receptor 1; CART: chimeric antigen receptor T cell; STAb: soluble T cell activating binder; JAK: Janus kinase; STAT: signal transducer and activator of transcription; CRLF2: cytokine receptor-like factor 2; TSLPR: thymic stromal lymphopoietin receptor; ALL: acute lymphoblastic leukemia; TNFSF4: tumor necrosis factor superfamily member 4; OX40L: OX40 ligand; AML: acute myeloid leukemia; B7-H3: B7 homolog 3; CAR-NK: chimeric antigen receptor natural killer cell; sIL-15: soluble Interleukin-15.

### Acute leukemia PDX model in other novel targets

Beyond the therapeutic investigations discussed earlier, the PDX model is crucial in evaluating natural extracts, nanocarriers, nanomaterials, metabolic interventions, and other signaling pathway targets [Table 5]. These emerging therapies represent the future promise for identifying potential targets, achieving precise drug delivery, enhancing therapy sensitivity, and, more importantly, overcoming resistance in innovative ways.

**Table 5:** Novel targets and other therapies tested in the PDX model

**Ref.**	**Category**	**Drug name**	**Key observations**
[182]	Targeting metabolic pathway	IACS-010759	IDHmi modulates metabolic pathways and induces leukemia cells to become reliant on mitochondrial function. IACS-010759 inhibits mitochondrial complex I, blocking the OXPHOS process. Combined treatment with IDHmi and OXPHOS inhibitors can overcome IDHmi resistance
[183]	Targeting metabolic pathway	Eganelisib	Residual leukemia cells after cytarabine treatment develop a PI3Kγ dependency. Eganelisib selectively inhibits PI3Kγ, thereby phosphorylating the atypical substrate PAK1, which impacts mitochondrial oxidative phosphorylation and overcomes chemotherapy resistance in leukemia cells
[184]	Targeting metabolic pathway	BAY2402234	In B-ALL cells, the activated PI3K/mTOR signaling pathway makes pS6+ cells rely on glucose for uridine synthesis. As the rate-limiting enzyme in de novo uridine synthesis, DHODH inhibition blocks uridine synthesis and sensitizes B-ALL to chemotherapy
[185]	Novel delivery method	Cas-CMV@LM	Adopting cell membrane vesicles derived from bone marrow MSCs overexpressing CXCR4 to target bone marrow. Subsequently, target AML cells through CD33 and CD123 aptamers anchored on the vesicles
[186]	Novel delivery method	Sora-MNC	The HA layer on the surface of Sora-MNCs achieves targeted accumulation in the bone marrow by recognizing the HARE on the bone marrow sinusoidal endothelial cells
[187]	Novel delivery method	αCD33-mAB-P/P	The αCD33-mAb-P/P nanocarrier system specifically delivers siRNA to CD33+ AML cells and enables precise RNA interference
[188]	Targeting pro-survival signaling	BCI-HCl	DUSP6 expression activates pS6 and pSTAT3, thus enhancing the cell proliferation and survival signaling. Targeting DUSP6 using BCI-HCl overcomes this resistance and demonstrates synergy with ruxolitinib
[189]	Targeting pro-survival signaling	P22077	USP7 inhibition increases the ubiquitination level of CHK1, and the CHK1 protein level decreases, impeding DNA replication fork progression. This sensitizes AML to chemotherapy
[190]	Inducing lysosomal cell death	Tetrandrine	Inhibition of TPC2 causes changes in lysosomal morphology and dysregulation of proteins related to lysosomal stability, making lysosomes vulnerable and inducing lysosomal cell death. This also promotes the accumulation of chemotherapeutic drugs in the cell nucleus, increasing sensitivity to chemotherapeutic agents
[191]	Antibody	Rosmantuzumab	The anti-RSPO3 antibody inhibits the RSPO3-LGR4 signaling pathway by blocking the interaction between RSPO3 and the LGR4 receptor, subsequently interfering with the activation of the Wnt1/β-catenin signaling pathway, which is a crucial pathway for maintaining the self-renewal and proliferation of LSCs
[192]	Cereblon E3 ligase modulator	CC-90009	CC-90009, a novel cereblon E3 ligase modulator, selectively degrades GSPT1 protein by hijacking the CRL4^CRBN^ E3 ubiquitin ligase complex, ultimately inducing apoptosis
[193]	Diet	Dietary methionine starvation	The deprivation of methionine through dietary intervention affects the metabolism and epigenetic markers of AML, particularly reducing H3K36me3 methylation
[194]	Novel target	Imetelstat	Imetelstat specifically binds to the RNA TERC, thereby inhibiting telomerase activity. This sensitizes AML to chemotherapy
[195]	Novel target	Ipatasertib	Selinexor activates PI3Kγ-dependent AKT signaling in AML. Inhibiting this pathway with ipatasertib, an AKT inhibitor, overcomes selinexor resistance in AML

PDX: Patient-derived xenograft; IDHmi: isocitrate dehydrogenase inhibitor; OXPHOS: oxidative phosphorylation; PI3Kγ: phosphoinositide 3-kinase gamma; PAK1: p21-activated kinase 1; mTOR: mammalian target of rapamycin; pS6: phosphorylated ribosomal protein S6; DHODH: dihydroorotate dehydrogenase; CXCR4: CXC chemokine receptor type 4; HA: hyaluronic acid; HARE: hyaluronic acid receptor; mAB: monoclonal antibody; siRNA: small interfering RNA; DUSP6: dual-specificity phosphatase 6; USP7: ubiquitin-specific protease 7; CHK1: checkpoint kinase 1; TPC2: two-pore channel 2; RSPO3: R-spondin 3; LGR4: leucine-rich repeat-containing G protein-coupled receptor 4; Wnt1: Wingless-type/Integration-1; LSCs: leukemia stem cells; GSPT1: G1 to S phase transition protein 1; CRL4^CRBN^: Cullin 4-RING E3 ubiquitin ligase complex containing Cereblon; TERC: telomerase RNA component; Akt: protein kinase B.

## CONCLUSION

Over the past few decades, significant strides have been made in unraveling the genomic landscape of acute leukemia, leading to the approval of numerous therapeutic agents. The array of heterogeneous pathogenic variants among patients has posed challenges in precisely targeting acute leukemia and combating drug resistance. Moreover, the complexities of acute leukemia diversity and the dynamic evolution within extensive subclones contribute to the emergence of drug resistance in clinical settings, underscoring the imperative for more individualized treatment. In this context, preclinical models, notably PDXs, have emerged as invaluable tools. By closely mimicking the clinical manifestations of acute leukemia and preserving the genetic diversity of acute leukemia cells, PDXs offer a platform for conducting more clinically pertinent evaluations, thereby facilitating further clinical testing and advancing our ability to tailor therapies to counter resistance.

The acute leukemia PDX models, immune-deficient mice transplanted with LSCs, are considered the optimal model for retaining the original characteristics of acute leukemia patients, including genetic features, clonal dynamics, evolution of genetic architecture, response to treatment, and the bone marrow microenvironment, which makes them highly valuable for acute leukemia research. Many researchers restricted passages in the PDX model to ensure greater biological and genomic similarity to the original leukemia samples, while serial passages, commonly used in developing large biobanks of acute leukemia PDX models, offer insights into genetic architecture evolution and aid in screening dominant clones. As previously mentioned, the availability of PDXs allows for the investigation of various therapies, including chemotherapy, targeted therapy, immunotherapy, and other novel therapies. Humanized PDXs have demonstrated potential in evaluating immunotherapy. Furthermore, as researchers are developing PDX biobanks, the PDX model provides a platform for screening potential therapies and discovering novel biomarkers. To model drug resistance, drug pressure selection or resistance patient samples are used, enabling the identification of the efficacy of combined agents or novel targets to combat drug resistance.

Although PDXs exhibit distinct advantages when conducting research on patient-derived samples, their application requires careful consideration of experimental suitability. Primarily positioned as preclinical models to provide translational evidence, PDXs are most appropriately employed following preliminary *in vitro* investigations, particularly in the evaluation of novel therapeutic agents, thereby mitigating excessive preclinical costs. Notably, PDX may not constitute the optimal platform for all pharmacological validations. Cell line models maintain critical utility in mechanistic studies targeting specific molecular pathways or phenotypes, especially for rare targets with limited patient sample availability or requiring precise genetic manipulation.

As for the microenvironment investigations, researchers have employed PDXs to study LICs and the microenvironment interactions that provide profound insights for resistance mechanisms, but there are inherent limitations for conventional PDXs. Murine-derived endothelial cells typically replace human stromal components, and interspecies metabolic disparities (e.g., hypoxia gradients, lactate concentrations) may compromise outcome accuracy, meaning that other *in vitro* models may be better substitutes for microenvironment research. The immunotherapies, such as ICEs, CAR-T, and ADCs, are different and frequently evaluated in PDXs because they directly target leukemia cells and are free from the incomplete replication of organ functionality and cytokine networks. Moreover, the co-transplanted healthy immune cells and peripheral blood mononuclear cells (PBMCs) can serve as supplementary components and enable PDXs for immunotherapy preclinical evaluations and safety assessments. However, evaluation in traditional PDXs still suffers from the absence of human immunosuppressive networks and may yield an overestimated efficacy of immunotherapy. Furthermore, murine models lack the capacity to recapitulate clinical treatment tolerability profiles. Critical pharmacological challenges, including drug-drug interaction potential during combination therapies and dose tolerance thresholds in patients with severe clinical comorbidities, remain inadequately addressed in PDXs.

## DECLARATIONS

### Copyright

© The Author(s) 2025.
