# Supplementary material for: Advances in the application of patient-derived xenograft models in acute leukemia resistance
Source: Cancer Drug Resist. 2025 May 28;8:23. doi: 10.20517/cdr.2025.18 (PMC12159603; doi:10.20517/cdr.2025.18)
Supplement: Supplementary file 1 [file cdr-8-23-SupplementaryMaterials.pdf]

Supplementary Materials

Advances in the application of patient-derived xenograft models in acute leukemia resistance

Ronghao Qin, Yuxing Liang, Fuling Zhou

Department of Hematology, Zhongnan Hospital of Wuhan University, Wuhan 430072, Hubei China.

Correspondence to: Prof. Fuling Zhou, Department of Hematology, Zhongnan Hospital of Wuhan University, No. 169 Donghu Road, Wuchang District, Wuhan 430072, Hubei, China. E-mail: zhoufuling@whu.edu.cn

Factors contributing to the establishment of patient-derived xenograft models for acute leukemia

Pretreatment

Pretreatment in mice is primarily intended to eliminate the hematopoietic system in order to promote engraftment, and it mainly consists of whole-body radiation and cytotoxic chemical treatment. Irradiation is widely adopted but often leads to a range of side effects, such as tissue damage and bone marrow suppression. Researchers found that this "side effect" improved the establishment of the human immune system. However, unirradiated mice maintained good health and still exhibited compatible engraftment of human immune cells [1]. Additionally, the effect of pretreatment may depend on leukemia subtypes. One study reported that irradiation significantly improved the engraftment of non-t (4;11) precursor B-cell acute lymphoblastic leukemia (pre-B ALL), while t(4;11) pre-B ALL could be effectively engrafted without pretreatment. Additionally, compared with irradiation, bone marrow sublethal conditioning with busulfan has equal efficiency for the xenotransplantation of acute myeloid leukemia (AML) cell lines [2].

Injection sites

The application of injection techniques elicits additional uncertainty. The intravenous injection is a widely used transplantation method thanks to its simplicity and practicability [3]. Nonetheless, intravenous injection requires larger cell counts compared with intrafemoral injection [4]. Intrafemoral injection is an orthotopic transplantation method that replicates the natural environment of the primary tumor and recapitulates the development of acute leukemia in the bone marrow and its dissemination throughout the body. Furthermore, intrafemoral injection in AML patient-derived xenografts (PDXs) tends to result in a relatively stable growth pattern and predictable overall survival [5], with a more convincing accuracy in predicting clinical response [6]. For PDXs of acute leukemia, subcutaneous injection is less frequently mentioned, partially because leukemia cells remain primarily confined to the injection site, with minimal infiltration into the peripheral blood and bone marrow, resulting in a contrasting growth pattern [5]. Nonetheless, subcutaneous injection provides valuable insights into the impact of drugs on local tumor growth, simulating the pathological features of the skin [7,8].

Immunodeficient mouse strain

The construction of PDX models is made more accessible by the availability of greatly improved immunodeficient mice (Figure 1). *Foxn1*(nu/nu) mice (nude mice), with a dysfunctional thymus, were the first employed for PDX model construction [9]. However, the initial attempts to transplant leukemia cells into nude mice were unsatisfying due to the presence of other immune cells such as B cells, NK cells, and macrophages [10]. This led to the establishment of severe combined immune deficiency (SCID) mice, which had a recessive mutation in the *PRKDC* gene encoding DNA-dependent protein kinase, resulting in a lack of normally functioning T and B lymphocytes [11]. To further deal with the presence of normal NK cells, macrophages, and lymphokine-activated killer cells, as well as leakage in a small percentage of older SCID mice [12], Lenny Shultz's team developed non-obese diabetic/SCID (NOD/SCID) mice by performing a backcross between C.B17-SCID mice and NOD mice, who achieved both T and B cell deficiencies of SCID mice and partial dysfunctions of the innate immune system of NOD mice [13]. The development of SCID mice and NOD/SCID facilitated further study of leukemia stem cells (LSCs) [14-16].

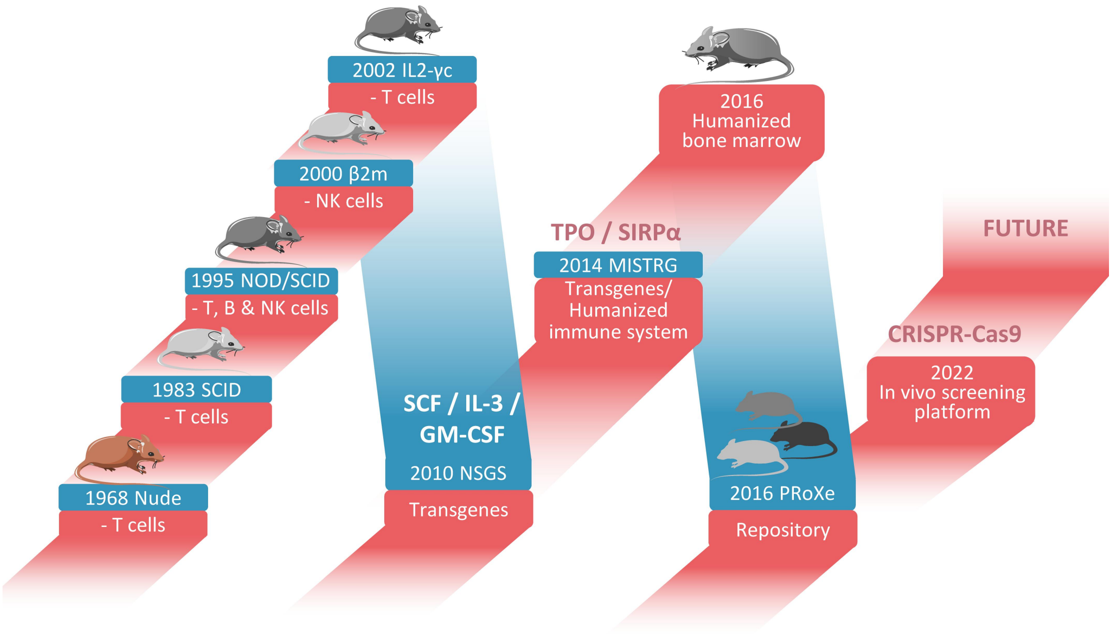

Supplementary Figure 1. A brief history of the development of PDX mice. The nude mice were developed in 1968, with a loss of T cell function. The following SCID mice and NOD/SCID mice achieved a broader range of immunodeficiency. Further depletion of the major histocompatibility complex class I beta2-microglobulin (β2m) gene and the gamma-common chain (γc) in the interleukin-2 (IL-2) receptor disrupts key signaling pathways of different immune cells and improves the immunodeficiency. In 2010 and 2014, human transgenes (stem cell factor, granulocyte-macrophage colony-stimulating factor, interleukin-3, thrombopoietin, and signal regulatory protein alpha) were introduced to the fully developed immunodeficient mice. Notably, the MISTRG also enabled the reconstitution of the human immune system. In 2016, researchers improved the engraftment of acute leukemia by leveraging a humanized bone marrow ossicle xenotransplantation model. Additionally, researchers envisaged evaluating therapeutic activity using adequately powered trials of PDXs and established a large, publicly available repository of ALL and AML PDXs (PRoXe). In 2022, an in vivo screening platform based on CRISPR-CAS9 and PDXs was used for acute leukemia target screening. For further development, the future belongs to you and me. *Adapted from Smart Servier Medical Art (https://smart.servier.com), licensed under CC BY 4.0; annotations and color modifications were added by the authors using Adobe Illustrator 2024.*

To elevate the engraftment rate, researchers tried various strategies, such as cytokine injection, human growth factor transgenes, and further immunodeficient modifications. One notable approach involves genetically modified NOD/SCID mice overexpressing human growth factors like stem cell factor (SCF), granulocyte-macrophage colony-stimulating factor (GM-CSF), and interleukin-3 (IL-3), which foster a more conducive in vivo environment for AML engraftment [17]. Moreover, researchers found that the elimination of NK cells before transplantation improved engraftment efficiency in NOD/SCID mice [18,19]. Thus, to further mitigate residual NK cell interference, NOD/SCID mice with a deletion of the major histocompatibility complex class I beta2-microglobulin gene (N/S-b2m-/-) were developed [17]. An alternative approach is NOD-$Prkdc^{scid}Il2rg^{-/-}$ (NSG) mice, with a gamma-common chain (γc) deficiency in the IL-2 receptor [20]. Notably, NSG mice faithfully replicated key pathological characteristics of advanced human leukemia, including organ enlargement, weight loss, and bone marrow fibrosis, underscoring their value in modeling human AML [21]. Consequently, mice harboring IL-2 receptor γc deficiency have been an excellent choice for establishing acute leukemia PDXs [6,22].

Further refinements on immunodeficient mice

NSG mice have been widely utilized not only for modeling diverse acute leukemia subtypes and drug testing but also as a foundation for subsequent refinements. Researchers further advanced this model by developing the NOD-$Prkdc^{scid}Il2rg^{-/-}$ SGM3 mouse (NSGS), which combined human cytokine expression and IL2RG knockout in the NOD/SCID background [23]. The NSGS mice allowed for rapid and stable evaluation of the engraftment of leukemia samples across different risk levels [24]. Additionally, NSG mice with null alleles for the major histocompatibility complex class I β2m, referred to as NSG-β2m, exhibited improved engraftment of AML and ALL compared with NSG mice [3,25]. As for the in vivo study of human innate immune cells, the genetically modified $Rag2^{-/-}Il2rg^{-/-}$ mice were developed, known as MITRG and MISTRG. They expressed various human cytokines and signal regulatory protein alpha (SIRPα) [1], and demonstrated remarkable engraftment rates of favorable-risk leukemia subtypes like inv(16) AML [26]. Furthermore, the BALB/c-$Rag2^{-/-}Il2rg^{-/-}SIRP\alpha^{hu/hu}$ BRG mouse strain humanized with SIRPα was crucial in identifying the robust self-renewal potential of LSCs [27].

The evolving establishment process, immunodeficient mice, and human transgenes have significantly enhanced the engraftment rates of acute leukemia samples, enabling the construction of PDX models that embrace diverse risk strata and acute leukemia subtypes and mirror their clinical response. Existing mouse models like NSG mice have demonstrated impressive engraftment rates and emerged as pivotal platforms for numerous translational research endeavors. Looking ahead, the next generation of acute leukemia PDX models is likely to emphasize further refinement and target specific leukemia subtypes to enhance their clinical relevance.

REFERENCES

1. Rongvaux A, Willinger T, Martinek J et al. Development and function of human innate immune cells in a humanized mouse model. *Nat Biotechnol* 2014;32:364-72. [DOI: 10.1038/nbt.2858]

2. Saland E, Boutzen H, Castellano R et al. A robust and rapid xenograft model to assess efficacy of chemotherapeutic agents for human acute myeloid leukemia. *Blood Cancer J* 2015;5:e297. [DOI: 10.1038/bcj.2015.19]

3. Gopalakrishnapillai A, Kolb EA, Dhanan P et al. Generation of Pediatric Leukemia Xenograft Models in NSG-B2m Mice: Comparison with NOD/SCID Mice. *Front Oncol* 2016;6. [DOI: 10.3389/fonc.2016.00162]

4. Paczulla AM, Dirnhofer S, Konantz M et al. Long-term observation reveals high-frequency engraftment of human acute myeloid leukemia in immunodeficient mice. *Haematologica* 2017;102:854-64. [DOI: 10.3324/haematol.2016.153528]

5. Schueler J, Greve G, Lenhard D et al. Impact of the Injection Site on Growth Characteristics, Phenotype and Sensitivity towards Cytarabine of Twenty Acute Leukaemia Patient-Derived Xenograft Models. In, editor^editors. Cancers; 2020. (ISBN No. 2072-6694)

6. Li J, Chen H, Zhao S, Wen D, Bi L. Patient-derived intrafemoral orthotopic xenografts of peripheral blood or bone marrow from acute myeloid and acute lymphoblastic leukemia patients: clinical characterization, methodology, and validation. *Clin Exp Med* 2023;23:1293-306. [DOI: 10.1007/s10238-022-00884-3]

7. Jia Y, Liu W, Zhan HE et al. Roles of hsa-miR-12462 and SLC9A1 in acute myeloid leukemia. *J Hematol Oncol* 2020;13:101. [DOI: 10.1186/s13045-020-00935-w]

8. Luo QY, Raulston EG, Prado MA et al. Targetable leukaemia dependency on noncanonical PI3Kγ signalling. *Nature* 2024;630. [DOI: 10.1038/s41586-024-07410-3]

9. Pantelouris EM. Absence of thymus in a mouse mutant. *Nature* 1968;217:370-1. [DOI: 10.1038/217370a0]

10. Nara N, Miyamoto T. Direct and serial transplantation of human acute myeloid leukaemia into nude mice. *British Journal of Cancer* 1982;45:778-82. [DOI: 10.1038/bjc.1982.120]

11. Bosma GC, Custer RP, Bosma MJ. A severe combined immunodeficiency mutation in the mouse. *Nature* 1983;301:527-30. [DOI: 10.1038/301527a0]

12. Sawyers CL, Gishizky ML, Quan S, Golde DW, Witte ON. Propagation of human blastic myeloid leukemias in the SCID mouse. *Blood* 1992;79:2089-98. [DOI: 10.1182/blood.V79.8.2089.2089]
13. Shultz LD, Schweitzer PA, Christianson SW et al. Multiple defects in innate and adaptive immunologic function in NOD/LtSz-scid mice. *J Immunol* 1995;154:180-91. [DOI: 10.4049/jimmunol.154.1.180]
14. Bonnet D, Dick JE. Human acute myeloid leukemia is organized as a hierarchy that originates from a primitive hematopoietic cell. *Nat Med* 1997;3:730-7. [DOI: 10.1038/nm0797-730]
15. Lapidot T, Sirard C, Vormoor J et al. A cell initiating human acute myeloid leukaemia after transplantation into SCID mice. *Nature* 1994;367:645-8. [DOI: 10.1038/367645a0]
16. Cox CV, Martin HM, Kearns PR, Virgo P, Evely RS, Blair A. Characterization of a progenitor cell population in childhood T-cell acute lymphoblastic leukemia. *Blood* 2007;109:674-82. [DOI: 10.1182/blood-2006-06-030445]
17. Feuring-Buske M, Gerhard B, Cashman J, Humphries RK, Eaves CJ, Hogge DE. Improved engraftment of human acute myeloid leukemia progenitor cells in beta 2-microglobulin-deficient NOD/SCID mice and in NOD/SCID mice transgenic for human growth factors. *Leukemia* 2003;17:760-3. [DOI: 10.1038/sj.leu.2402882]
18. Yoshino H, Ueda T, Kawahata M et al. Natural killer cell depletion by anti-asialo GM1 antiserum treatment enhances human hematopoietic stem cell engraftment in NOD/Shi-scid mice. *Bone Marrow Transplantation* 2000;26:1211-6. [DOI: 10.1038/sj.bmt.1702702]
19. Notta F, Mullighan CG, Wang JC et al. Evolution of human BCR-ABL1 lymphoblastic leukaemia-initiating cells. *Nature* 2011;469:362-7. [DOI: 10.1038/nature09733]
20. Ito M, Hiramatsu H, Kobayashi K et al. NOD/SCID/γcnull mouse: an excellent recipient mouse model for engraftment of human cells. *Blood* 2002;100:3175-82. [DOI: 10.1182/blood-2001-12-0207]
21. Sanchez PV, Perry RL, Sarry JE et al. A robust xenotransplantation model for acute myeloid leukemia. *Leukemia* 2009;23:2109-17. [DOI: 10.1038/leu.2009.143]
22. Wang K, Sanchez-Martin M, Wang X et al. Patient-derived xenotransplants can recapitulate the genetic driver landscape of acute leukemias. *Leukemia* 2017;31:151-8. [DOI: 10.1038/leu.2016.166]

23. Wunderlich M, Chou FS, Link KA et al. AML xenograft efficiency is significantly improved in NOD/SCID-IL2RG mice constitutively expressing human SCF, GM-CSF and IL-3. *Leukemia* 2010;24:1785-8. [DOI: 10.1038/leu.2010.158]
24. Díaz de la Guardia R, Velasco-Hernandez T, Gutiérrez-Agüera F et al. Engraftment characterization of risk-stratified AML in NSGS mice. *Blood Advances* 2021;5:4842-54. [DOI: 10.1182/bloodadvances.2020003958]
25. Kollet O, Peled A, Byk T et al. β2 Microglobulin-deficient (B2mnull) NOD/SCID mice are excellent recipients for studying human stem cell function. *Blood* 2000;95:3102-5. [DOI: 10.1182/blood.V95.10.3102]
26. Ellegast JM, Rauch PJ, Kovtonyuk LV et al. inv(16) and NPM1mut AMLs engraft human cytokine knock-in mice. *Blood* 2016;128:2130-4. [DOI: 10.1182/blood-2015-12-689356]
27. Jinnouchi F, Yamauchi T, Yurino A et al. A human SIRPA knock-in xenograft mouse model to study human hematopoietic and cancer stem cells. *Blood* 2020;135:1661-72. [DOI: 10.1182/blood.2019002194]
